# Applying a new concept of embedding qualitative research: an example from a quantitative study of carers of people in later stage dementia

**DOI:** 10.1186/s12877-019-1240-x

**Published:** 2019-08-22

**Authors:** Michele Abendstern, Karen Davies, Helen Chester, Paul Clarkson, Jane Hughes, Caroline Sutcliffe, Fiona Poland, David Challis

**Affiliations:** 1 Personal Social Services Research Unit, University of Manchester, Manchester, UK; 2 School of Biological Sciences, University of Manchester, Manchester, UK; 3 Formerly Personal Social Services Research Unit, University of Manchester, Manchester, UK; 4 School of Health Sciences, University of East Anglia, Norwich, UK; 5 Institute of Mental Health, University of Nottingham, Nottingham, UK

**Keywords:** Embedded study, Qualitative methods, Dementia, Carers, Expressed experiences

## Abstract

**Background:**

Qualitative methods are increasingly included in larger studies to provide a richer understanding of people’s experience. This paper explores the potential of using a novel approach to embedded qualitative design as part of an observational study examining the effectiveness of home support for people in later stage dementia in England. The method involved collecting and analysing unsolicited conversational comments made by participants as they completed standardised measures. An evaluation of the method is presented using the voices of participants to illustrate its potential.

**Methods:**

The conversations of 17 carers recruited to an observational study were audio recorded to gather commentary made while completing a structured interview. Data were interrogated using thematic analysis to investigate the feasibility of conducting an embedded qualitative study, the potential richness of the material and participants’ reactions to formal questioning and participating in research.

**Results:**

The findings revealed that qualitative data were available from this approach. Analysis generated three themes from carers: conflicting carer emotions; the importance of maintaining normality and agency within day-to-day life; and tensions between these desires and making use of formal services. Important issues for carers were revealed establishing the benefit of using the method. The advantages of exploiting unsolicited conversation included enhancing understanding of people’s lived experience, reducing participant burden in research and easing the process of data collection. In addition, it provided an opportunity to evaluate individuals’ experience of the research process.

**Conclusions:**

The findings demonstrate how unsolicited comments during structured interviews may appear incidental but can reveal important aspects of living with dementia. The method also emphasised methodological challenges for research in dementia, including the influence and impact of the research context. Further research is required to evaluate the method with other groups including people with dementia themselves.

**Electronic supplementary material:**

The online version of this article (10.1186/s12877-019-1240-x) contains supplementary material, which is available to authorized users.

## Background

*“Almost every simple question, you get some kind of story”*

(Research interviewer)

Mixed methods research offers the opportunity to combine the benefits of research approaches with the inclusion of qualitative methods extending the depth of understanding of people’s perceptions, experiences and feelings [1]. There are many different designs used for mixed methods [2] including an increasing interest in embedding qualitative studies within larger quantitative research [3]. However, to our knowledge, none have considered the naturally embedded data that occur through unsolicited conversation during structured interviews frequently used in quantitative studies. This paper reports the findings from an exploratory qualitative study using such data from carers of people in later stage dementia during their participation in a structured interview. The study drew upon ethnographic principles in relation to data collection approaches (the unobtrusive collection of data on usual life) to appreciate “the ways that people understand and account for their day-to-day situation” [4]. A larger observational quantitative study of the effects of different approaches to home care services for people with dementia and their carers provided the context for the embedded qualitative study [5].

This article has two aims: to demonstrate the value of the novel method of qualitative data collection and to illustrate how this can be employed to gain and analyse data from carers involved in structured interviews of people with dementia. More specifically, by gathering qualitative data during a single structured interview, that is, one which uses standardised quantitative measures, we aimed to explore whether comments embedded within them could facilitate a ‘sociological lens’ that could allow us to access the views of carers as expressed while they were engaged in completing standardised measures [5, 6]. In addition we consider whether the burden for families participating in research could be minimised by streamlining the research process through ‘doubling up’ on the data collection, avoiding the need for the separate collection of qualitative and quantitative data.

We begin by presenting the background to the study, considering existing evidence about dementia that motivated the wider research. We then describe the rationale for the development and use of the embedded method. Next, in order to illustrate and examine the utility of using incidental conversational data collected via the embedded qualitative method, we present key findings from the data analysis. The discussion considers how this addressed the two aims of the embedded study and the impact of the research process on participants revealed by the findings.

### Context of living with dementia: the carer perspective

Dementia is sometimes conceptualised within research, policy and practice as a problem: a drain on resources [7, 8], a burden on families and the state [9, 10], and a personal disaster for those who live with it [5, 11]. Research in this area often aims to quantify such problems using standardised measures such as quality of life instruments, measures of burden and costs of caring [12]. Whilst this may produce a familiar narrative which may represent the lives of some people living with dementia, there is a growing interest in finding ways to attend more closely to the ‘lived experience’ of carers who support them [13], which may contradict the conventional wisdom so frequently seen in the media. It may be that the tendency to represent caring for people with dementia in singularly bleak terms is at odds with carers’ own more nuanced and positive discourses [14].

This paper explores a novel embedded research method, capturing carers’ views when expressed as informal comments occurring naturalistically and unprompted while completing standardised measures in face-to-face interviews. The paper illustrates how these comments can offer more contextual access to a deeper understanding of the social world in which people in later stage dementia and their carers live, as they put into their own words their views of their world, including their experience of formal service provision at home [5]. A wealth of literature, both quantitative and qualitative, exists on carer burden. Far fewer studies have explored carer experience through observation of day-to-day life or how this is perceived by carers during unsolicited conversation.

## Method

### Rationale for an embedded qualitative study

This was not a typical, stand-alone, qualitative study. Rather it set out to evaluate the feasibility of gathering qualitative data made available during structured research interviews with carers and people with dementia taking place as part of a large quantitative study. Moreover, it was not a conventional embedded study design as it did not include additional data collection activities. The study adopted a novel approach based on the assumption that the conversation accompanying the completion of standardised measures within an interview setting would be specifically informative. We refer to this as an adapted, embedded, qualitative study of unsolicited conversation. Experience within the research team of undertaking structured interviews suggested that participants use conversation to comment on, qualify, and elaborate their answers during standardised interviews, despite the formal questioning structure. We proposed that these data could offer a value-added dimension, potentially enabling a fuller understanding of people’s expressed experiences. Traditional embedded studies have used related but parallel qualitative findings to interpret quantitative results. In contrast, we set out to explore whether ethnographic data relating to lived experience, could be gained from a quantitative research design involving structured interviews.

There were two obvious challenges in conducting the adapted embedded qualitative study. First, standardised measures use closed questions aiming to elicit factual responses that can be recorded using scaled items. These are not intended to encourage reflection or explanation of feelings. The adapted embedded qualitative study aimed to establish whether participants’ conversation within such a setting nonetheless generated rich and relevant insights into living with dementia. Second, these measures were administered by researchers in the field with items intended to be as standardised as possible, and so might limit the quantity and quality of unsolicited comments from carers and be subject to variation according to both interviewer and interviewee.

The larger observational study [5], which forms the context for the embedded approach, and the embedded study itself, had discrete research aims and methods (Table 1).

**Table 1:** Summary of research aims and processes for quantitative and adapted embedded qualitative studies

Study type	Study aim	Method	Sample	Analysis
Observational study	To explore the presence and effect of different approaches to home support for people in later stage dementia within selected geographically distinct areas in England.	Structured standardised interviews at 2 time points	< 300 people aged 60+ in later stages of dementia and their carers across 6 NHS Trusts in England^a^	Statistical
Adapted embedded study	To explore: 1. The feasibility of collecting rich contextual, data during structured interviews 2. Foregrounding the voice of carers during structured research interviews 3. The research process from perspective of participants	Audio recording of subset of follow-up (time 2) structured standardised interviews	Subset of 17 carers from one trust Purposive sample using PANT score	Thematic analysis of incidental conversational data

^a^Further details of inclusion criteria are provided below. Full study details: Chester et al [5]

### Recruitment to the main and embedded studies

Researchers recruited participant dyads to the larger study from community services in selected geographical areas, comprising six English NHS Trusts. Services were chosen from previous national surveys of home support services for people with dementia and their carers [15]. Dyads were a person in later stage dementia with their informal carer, who could be a spouse, relative, or friend. To be eligible to take part in the study individuals with dementia had to be aged 60 years or over, have an informal carer, and be receiving support at home or in the community. The stage of dementia characterising the condition of the person who was living with dementia was identified by a screening question that excluded those with mild or moderate dementia [5, 16]. Community services were asked to identify a cohort of eligible people in receipt of home support from their records.

Prospective participants were invited to take part by staff working within the community services. Those expressing interest were then sent an information sheet from the research team. Participants who chose to take part were interviewed at home by research staff from participating trusts. Participants met their interviewer for the first time at this point. Informed consent was obtained prior to the interview on the day [5].

A sub-sample taken from the larger study were used for the embedded study by inviting dyads from a single trust with a diverse socio-demographic profile to allow their structured interviews to be audio recorded. The Practitioner Assessment of Network Type (PANT) derived at baseline interview was used to guide purposive sampling for the sub-study [17, 18]. Three dyads from each of the five PANT types were recruited to ensure that the sample reflected a variety of patterns of social support. Purposive sampling was again undertaken to ensure participants included an equal number of male and female carers (Table 2). The final sample size was determined by data saturation: when no new themes arose from analysis of the data [19, 20]. The first five interviews produced 56 descriptive codes, the next five produced two more codes that could not be merged or linked to existing codes and a further five produced three new codes. A total of 17 dyads were recruited from a total of 23 original invitees (Table 2). The current analysis focuses specifically on data gathered from carers who were interviewed on their own where feasible.

**Table 2:** Dyad relationships, living situation, and gender

ID	Dyad features			Person with dementia	Carer
1	Spouses (*n* = 8)	Co-living(*n* = 10)	Male carer (n=5)	Wife	Husband
2				Wife	Husband
5				Wife	Husband
8				Wife	Husband
10				Female friend	Male friend
6			Female carer (*n* = 11)	Husband	Wife
14				Husband	Wife
16				Husband	Wife
4	Inter-generational (*n* = 9)			Mother	Daughter
17				Mother	Daughter
13		Live separately (*n* = 7)		Mother	Daughter
7				Aunt	Niece
9				Aunt	Niece
11				Mother-in-law	Daughter-in-law
12				Father	Daughter
15				Father	Daughter
3			Male carer (n=1)	Father	Son

### Data collection

The larger study involved carers completing a range of standardised measures (Table 3. The edited questionnaire is presented in a Additional file 1). These assessed carers’ health, their views of their relatives’ health, wellbeing and experience of support [5]. Structured interviews were undertaken at two time points 6 months apart by research staff from participating trusts. There was no additional data collection for the embedded study. Rather, structured interviews of the sub-set of 17 participants were audio recorded in order to document unsolicited commentary and conversation as the interview progressed. No additional questions were used and interviewers were instructed to neither prompt nor shut down conversation. Interviews with carers lasted between 20 and 80 min and were undertaken at the 6 month follow-up interviews within the larger study. Interviewees are referred to as participants in this paper and their relationship to the person with dementia within their dyad is noted where appropriate. This terminology is used deliberately to reflect the way carers identified themselves, rarely referring to themselves as ‘carers’ but, rather, as husbands, wives, daughters, sons and nieces.

**Table 3:** Measures used within structured interviews with carers

Person with dementia characteristics and circumstances	
-Demographic information (including gender, age, ethnicity, and marital status) -Living situation (home, hospital, or care home) -Practitioner Assessment of Network Type (PANT) [17] -Bristol Activities of Daily Living Scale (BADLS) [21]	
Person with dementia quality of life	
-Dementia Quality of Life scale (DEMQOL) – proxy version (30-item) [22]	
Carer characteristics, health and burden	
-Demographic information (including gender, age, ethnicity, marital status) -EQ-5D-5 L [23] -General Health Questionnaire (GHQ-12) [24] -Short Sense of Competence Questionnaire (SSCQ) [25] -Zarit Burden Interview (ZBI − 22-item) [26]	
Support to person with dementia	
-Informal support by carer (relationship to person with dementia; care tasks undertaken – nature and frequency; employment status) *Adapted from* [27]. -Formal care (health and social care services; adaptations and equipment; inpatient and outpatient care; and ambulance use) *Adapted from* [27] -Reliability, sufficiency and effectiveness of care (personal; daily household; and weekly household) [28]	

Adapted from [5]

In addition to the interview sample, a focus group (*n* = 5) with interviewers who had undertaken both recorded (adapted embedded qualitative study) and unrecorded (larger quantitative study) interviews provided an opportunity to corroborate findings. The interviewers were experienced research staff, often with professional experience of working with older people. All had completed Good Clinical Practice and training specific to this study. The discussion capitalised on their shared experience of conducting structured interviews [29] and drew on experiences beyond the embedded study sample. The focus groups took place at a mid-point in the interview data collection. Members were presented with early findings on participants’ views and asked to reflect on three issues: whether the themes ‘rang true’ to them, a recognised construct within qualitative research [30], and a means of validating findings; and whether they could identify anything missing from them, helping to establish data saturation. Finally, they were also asked about their experiences of undertaking the interviews. The quotation introducing the article is one response from this discussion.

### Data analysis

The interviews and focus group were professionally transcribed and then analysed by two of the authors (MA and KD). The analysis was supported by ATLAS. Ti 7.5 software which enabled coding and interpretation to be systematically organised and refined. A thematic analysis [31] aimed to reveal “windows on the participants’ social world, referring to and representing feelings, perceptions and events that exist apart from the data themselves” [32]. The analysis also explored the relationship between individuals’ responses and the questions in the standardised measures at an indicative level rather than providing a detailed comparison and is used here descriptively. The kind of questions prompting specific comments are referred to but a detailed comparison of the unsolicited conversation and questions was not undertaken for this study. The coding procedure was based on Flick and Gibbs’ [33] three stage process: (i): assigning descriptive codes summarising surface features of the data; (ii) generating categories linking areas of similarity and difference; (iii) identifying analytic codes to segment the data into abstract, theoretical concepts or themes. Analysis combined deductive and inductive practices. The former utilised a conceptual framework drawn from the literature [34–36] encompassing inter-related issues that provided three a priori themes to inform but not to limit data analysis:

i. Carers’ responses to and understanding of dementia;
ii. Relationship between formal support available and family needs;
iii. Perceptions of the quality of the support received.

The analysis also inductively identified new concepts from the data [19]. The process of moving from raw data to final themes was iterative with interview transcripts coded independently by both researchers and modified through discussion between researchers to create themes and subthemes. An example of this process is provided in Table 4.

**Table 4:** Example of thematic analysis

Step 1: Standardised question	Are they (formal support services) reliable or are there sometimes lapses or are they only reliable at certain times?			
Step 2: Participant response:	“We don’t have people on a Saturday and a Sunday because it … it was very hit and miss … I didn’t particularly want to get up at seven o’clock on a Saturday morning … … they caught me a couple of times halfway through a shower, which is horrible … There was one weekend when we waited for them to come and at half past 11 they hadn’t come so I said, forget it … So, … We get up on a Saturday now when we want to, well We … have breakfast in dressing gowns, sit and read the paper and then when I feel like it … go and shower … but why not!? We’re retired”			
Step 3: Thematic analysis process	Summary	i)Broad descriptive code	ii)Concepts/sub themes	iii)Themes
Step 3: Thematic analysis of data from summary to theme	Unreliability of weekend service leading to feeling out of control in own home. Cancelled service to regain this.	Formal service - domiciliary care (negative)	Formal service insensitive to circumstances. Control over home life at a cost	Maintaining normality Retaining agency

### Quality assurance

Trustworthiness and credibility, concepts used to assess the robustness of qualitative research [37], were achieved through careful team reflection and sense checking. Themes were developed by MA and KD from their close reading, summarising and synthesising of the data. This was initially undertaken individually with follow-up discussion firstly between MA and KD and then with the full research team who were provided with summary data and emergent themes. The latter were refined through these discussions with differences of opinion resolved via consensus.

Early findings and themes were also shared with a Public, Patient, and Carer Involvement (PPCI) group recruited to the main study [38]. This group consisted of present and former carers of those with dementia and also included one person with dementia. It had 13 members who met with the research group face-to-face in a series of regular meetings. They were recruited via Together in Dementia Everyday (TIDE) a national advocacy group for carers of those with dementia and through a previous programme of research. They had many years’ experience in supporting people with dementia and in advocating at a national level on support and policy for carers of those with dementia. To contribute to analysis of the embedded qualitative study data the researchers asked members the same questions as those used in the focus group, that is: whether the early findings ‘rang true’ and whether anything significant was missing. Responses from both groups were confirmatory with no new themes emerging.

## Results

The findings present the results of the analysis of the unsolicited conversational comments. Evaluation of the method is then considered in the discussion.

### What did the adapted embedded qualitative method reveal?

A rich and informative set of data was uncovered through recording and then analysing the conversational comments of carers during structured interviews. The depth and range of insights was surprising, given the formal nature of the structured interviews and is presented in some detail in this section with participants’ words quoted to illustrate the range and quality of data generated. The themes derived from the carers’ unsolicited comments throughout the interviews, data that would normally be unrecorded, revealed carers’ perceptions about important aspects of living with dementia. The structured interviews included questions about social contacts, relationships, feelings, everyday activities and experience of support. While carers sometimes directly related their comments to specific questions in the structured interviews, equally often, a particular theme could be derived from comments arising across the whole interview as participants chose to return repeatedly to express the same feelings or experiences irrespective of the question being formally asked at the time. Three main themes emerged from this type of data:

- Conflicting emotions of acceptance and feelings of anxiety, anger and guilt
- Maintaining normality and retaining agency
- Tensions between retaining agency and overseeing/integrating support

The themes connect and resonate with each other, focussing on descriptions of emotions, through articulating hopes and expectations for everyday life, to outlining implications of support. The researchers’ interpretation of participants’ responses was used to develop the relative notion of normality used here, as referring to the idea of the dyad’s way of life continuing, albeit with necessary adaptation to the changing characteristics of the person with dementia. Thus, there was no one standard formula for ‘normality’, rather, in each case, it reflected and expressed how carers defined their life, drawing on their pre-existing family culture [39]. Agency is defined as the capacity to be the director and author of one’s actions, to make decisions and choices based on one’s own will, and to do what one had intended [40]. Both concepts were articulated by carers in relation to the lives of individuals with dementia, their lives as carers, and, often, how they operated together as a dyad.

#### Conflicting emotions of acceptance, anxiety, guilt and anger

The structured interview questions included several items that asked carers to consider their views of their emotional state. Carers frequently qualified their answers as they tried to express the specific nature of their feelings. Comments such as *“it’s hard to answer these”* or *“sometimes it’s easy and other times it’s difficult”* revealed the challenge these questions could pose to carers, for defining how they felt about their situation. Carers’ discourse revealed extensive differences in the range and intensity of the emotional responses they expressed about caring for a relative with dementia. Many also reflected on how their emotional state varied from day to day (Table 5).

**Table 5:** Contrasting emotional responses experienced by carers

Main theme	Subtheme
Acceptance	Acceptance of changes relating to dementia Empathy and respect for person with dementia
Anxiety for the future	Anxiety about impact of changes due to dementia Guilt about not doing enough for person with dementia
Anger	Anger/frustration at changes related to dementia

##### Acceptance

When questions were asked directly about whether they felt any anger or embarrassment about how their relative responded or behaved, carers often answered by expressing their unreserved acceptance of their relatives’ behaviour and condition while conveying a fierce loyalty towards them. They often accompanied their stated readiness to accept their situation with words of optimism in the face of the challenges that dementia posed for the family. One wife, when asked whether she had recently felt that she couldn’t overcome her difficulties, explicitly commented that she regarded the difficulties of caring as “challenges” rather than difficulties. She believed she could manage or accept challenges without feeling she had to tax herself further:

*If something arises that isn’t working very well I can usually think through, to make it better, and if not, I just say, oh well, there’s not much I can do about it (Participant 6)*

Most carers described how caring changed their relationships, with a greater emphasis on their role in overseeing care arrangements, directly managing a range of caring tasks and taking responsibility for all the decisions in the household. Whilst some noted that these changes were also accompanied by loss, including loss of emotional support from their relative, others mentioned finding new activities that they could do together that enhanced their lives. Carers’ words often revealed a deep understanding of the challenges posed by dementia and expressed a desire to also broaden their knowledge of how dementia was affecting their relative. Many used words that conveyed empathy, respect and compassion for their relative.

*The hardest thing now is she says to me, I don’t want to be a burden. And well, she used to say that to me. Now she’s saying, I know I’m a burden … And I go, but you’re not a burden* (Participant 17)

##### Anxiety for the future

Participants referred to a heightened sense of anxiety but made a distinction between feelings of anxiety and depression. For example, when asked whether she felt anxious or depressed, one wife stated “*I wouldn’t say it was depression … just apprehensive”* (Participant 14). Uncertainty about the progress of dementia and managing future care needs were the predominant source of anxiety. A daughter commented that she worried “*constantly”* about the current and future situation, whilst another carer summarised his concerns, explaining, “*You know it’s going downhill”* (Participant 1). The increasing demands of caring were linked with comments about emotional exhaustion and a profound sense of responsibility for someone else’s life, as exemplified, *“(I) just worry about whether I can keep up with the phases (of dementia)”* (Participant 17). Many carers expressed anxiety about being the sole carer. They tended to link this to concerns about their own health, anticipating deterioration in what they were able to do for their relative. In response to a question about whether the care she provided was always reliable, one wife explained: “*It does worry me what’s going to happen in the future. If I fell ill he’ll have to go into care”* (Participant 14).

##### Guilt

Feelings of guilt were also expressed by several carers when asked whether they were doing enough for the person with dementia. Within this sample, this appeared to be more prominent in younger generation carers, particularly daughters (there was only one son in the sample). Comments included guilt about feeling they could have done more, whether they could continue as the primary carer much longer, and whether they should continue to go out to work rather than become a full time carer. For example:

*I do feel guilty about work … it’s not just the pressure of work … it’s the guilt when I go … I go in my mum’s room every morning before I go and then I go straight in there when I come home … But I hate going … I hate that feeling* (Participant 17).

##### Anger

Most participants did not elaborate their feelings of anger and provided no more than a hint of their frustration. For example, one man commented when asked whether his wife was worried about forgetting people’s names: “*I don’t think it bothers either of us, I mean I … get frustrated”* (Participant 5). In a few cases, negative feelings towards the person with dementia were more strongly expressed. For example, a husband (Participant 8) became cross with his wife when she could not answer a question for the researcher, asking: “*Is there a question about beating your wife?”* Occasionally, the quality of the relationship between the person with dementia and their carer appeared more markedly coloured by frustration and anger. Expressing her exasperation with her parents who refused any form of formal support, one daughter stated at the end of the interview:

*I get to boiling point … when my mum is ringing up and … she’ll be wailing and crying. My hands are tied and there’s nothing more I can do…. You just want to explode at times.* (Participant 12)

In summary, many carers expressed a high level of acceptance of their situation whilst also referring to considerable anxiety and even anger at other points. A minority expressed more negative feelings throughout. It might be that these were people whose relationships were fraught before the onset of dementia. However the data does not provide firm evidence on this issue.

#### Maintaining normality and retaining agency

Maintaining normality and retaining agency was identified from the data, revealing underlying features related to carers’ particular hopes and expectations for their lives. Responses used in the development of this theme came from a range of questions in the structured interview, including those about social contacts, activities of daily living, and worry about their quality of life (Table 6).

**Table 6:** Maintaining normality and retaining agency

Main theme	Subtheme
Maintaining normality	Sustaining relationships, roles and social engagement
Retaining agency	Participation in meaningful activity Promoting independence

##### Maintaining normality

A desire for normality was expressed through remarks relating to accommodating changes in relationships, roles and sustaining social engagement. Carers showed a determination to maintain the quality of their relationships despite changes in their roles within those relationships. For example, one husband (Participant 5) referred to how he continued to ask his wife’s opinion about buying clothes, as he had always done, despite changes in other aspects of their domestic life. Carers frequently referred to their relatives’ talents and interests before dementia, often presenting these as a way of prolonging a sense of ‘life as usual’.

Some carers reported prioritising opportunities for social engagement, supporting their relative with dementia to continue to take part in activities they enjoyed. Maintaining normality for carers was seen as aided by friends and relations who understood the implications of dementia. Examples were cited where family friends were regarded as ‘dementia aware’ and were content to participate in social activities with the person with dementia and carer together. Others spoke of friends who took the carer’s responsibilities into consideration when making arrangements with them.

Nevertheless, social contacts were regarded as difficult to maintain in practice. Social contacts for the person with dementia themselves dwindled beyond the immediate family, as outings became difficult and fewer friends visited. One daughter-in-law stated at the end of the interview (when asked if there was anything she’d like to add) that she thought her mother’s wellbeing was largely due to continued social connections: “*We feel that that’s key to her longevity and … her … pretty good quality of life … all things considered”* (Participant 11). There were carers who referred to ‘normal life’ as ‘on hold’. This was expressed in terms of deterioration in the conversations between relatives and decline in both their own and their relatives’ social life. One husband described conversation as *“plateauing [at] round about the thirty year old”* referring to the things his wife could remember (Participant 1). Some described themselves as simply being too exhausted to continue with past activities alongside having few opportunities to go out.

##### Retaining agency

This theme is considered here in relation to how carers sought and found ways of supporting the person with dementia to do things for themselves (directing their own actions) and to participate in meaningful activity. Remarks reflecting this theme came from across the interviews but in particular were located in responses to questions about activities of daily living and quality of life. Examples from carers’ discourses included providing opportunities for the person with dementia to continue routine tasks independently, such as food preparation, showering, shopping, or even getting out of a chair. One daughter described how she had learnt to support her father’s independence in relation to his personal care through buying products that he recognised:

*He kept going down to the kitchen asking me ‘what’s this?’ …*. *And I realised that I had to take a step back and buy things that he would recognise* (Participant 15).

Participation in meaningful activity was recognised by carers as an important factor contributing to personal wellbeing. Examples included encouraging continued participation in lifelong interests and hobbies. One daughter described taking her mother, a talented knitter, to a ‘knit and natter’ club where she made baby blankets for friends. This was perceived by her daughter as providing her with a sense of usefulness to others, alongside companionship, something she sensed was of continued importance to her.

Supporting or promoting agency in a relative was not always presented as straightforward. There were examples of differences in opinion between the person with dementia and their relative about what was in their best interests. For example, a niece described her unsuccessful attempts to encourage her aunt to attend a luncheon club to relieve her isolation. This suggests that the person with dementia also expressed a determination to make decisions for themselves and retain their own agency, albeit at odds with their carer.

#### Tensions between retaining agency and overseeing/integrating support

All carers expressing a view on receiving care stated a firm preference for support that linked with their desire for normality and agency. However, they cited examples of tension between retaining this hope and integrating support from external agencies into their everyday lives. Their comments were largely located in addressing three measures in the structured interviews relating to activities of daily living and quality of life (Table 7).

**Table 7:** Carers’ views of the interface between carers and formal support

Main theme	Subtheme
Maintaining control in the home	Taking the lead role in arranging formal care Reliable and trustworthy formal support
	Continuity of staff
Working in partnership with health and social care organisations and professionals	Coordinated support rather than fragmented support Compassionate support (sensitive, communicative, relational)
Benefit of finding carers from own network	Neighbours and friends as carers

##### Maintaining control in the home

Carers described a diverse assortment of support arrangements including the involvement of other family members and blending formal and informal care. Satisfaction was expressed where carers felt they had developed a partnership with staff from the care agency whom they trusted and were able to negotiate support, integrate it with their personal and home life, and observe positive relationships between the staff and person with dementia. However, carers also reported that services could hinder their desire for normality and agency.

Some carers’ comments suggested that external care could be intrusive and so disrupted the household’s routine. For example, a husband, when asked how reliable he thought the carers were, reported that the domiciliary care was reliable during the week but *“diabolical”* at the weekends, regularly turning up late which interfered with their plans for the day.

*Weekends can be a nightmare … on Sunday, for example … I wanted to go to church, um, and I waited and I waited. … rang and rang and rang, and … it was well after 10:00* (Participant 5).

Carers described a trade-off between external support and retaining a lifestyle they preferred, sometimes choosing to cancel services that did not suit them. They expressed this as a lack of flexibility, where arrangements did not ‘fit’ with the individual and lacked staff continuity:

*I do get a little bit … angry with the care organisation when they send me different people in a week because it’s quite important that I am able to hand over. D needs the continuity, he needs to know who is coming* (Participant 6).

##### Working with care agencies and professionals

Dealing with the array of services and professionals could be confusing for families and many struggled to know who they had seen. Carers intimated that they sometimes felt let down by formal services who failed to engage personally with them and/or the person with dementia. These were described through examples of poor communication and insensitive interactions from professionals. Experiences reported by carers included no clear follow-up after discharge from specialist assessment, perceiving their views to be ignored by professionals when they described the person with dementia’s symptoms, physical health conditions being overlooked by health professionals who only ‘saw’ the dementia or alternatively a lack of sensitivity around how dementia might affect someone. Reflecting on an appointment with a dietician one husband stated:

*It didn’t go too well with S because there was no response, there was nothing. But she (dietician) insisted on doing it* (Participant 1).

Carers expressed satisfaction when formal care workers developed a relationship with the person with dementia, in contrast to focusing on care tasks only. A high level of commitment from individual workers was noted by some participants. For example, one son, responding to a question about how long the formal carer who looked after his father stayed, commented:

*He comes even when he doesn’t have, you know … Yeah, the chap’s all right. He does it beyond the call of duty, so to speak* (Participant 3).

##### Finding carers from own network

In several cases, carers had found their own care staff using their social networks, so that paid carers were people already known to the family/dyad. Carers were positive about these arrangements often describing them as providing more than they expected. The personal connection with the carer and person with dementia also meant that these paid carers were perceived as operating in a different space, somewhere between carer and friend. A husband stated *“she’s very good”* when recalling everything his wife’s paid carer did and then explained a link that he believed helped the relationship:

*Strangely enough she was er, one of the children S used to cross as the lollipop lady so there’s that link. That’s why we use her here because, you know, S remembered her* (Participant 1).

## Discussion

This paper has described the development of a method, tailored to the context of the wider study, and then evaluated its contribution using the findings from carers involved in the study to illustrate that findings from larger studies can be specifically contextualised and enriched without increasing participant burden. It has illustrated the extent and depth of data generated from unsolicited comments during structured interviews, endorsing the use of an adapted embedded qualitative method in the context of interviewing carers of people in the late stage of dementia. In the words of an interviewer (focus group member): *“Almost every simple question, you get some kind of story”*. The discussion considers firstly, carers’ perspective of living with dementia revealed during structured interviews and secondly, an evaluation of the adapted embedded qualitative method.

### Perspectives of living with dementia revealed during structured interviews

The method generated findings about carers’ experiences that resonate with the literature. Whilst there is much qualitative evidence about carer experiences, these specific findings nevertheless illustrate deeply-held themes, in the carers’ own words stimulated by commonly-used questions in standardised research, prompting their sometimes contrasting and qualifying comments on how they view and want to live their lives. The unsolicited comments and conversations during structured interviews revealed a more active consideration by participants of the issues raised and the language used within the standardised questions.

Structured interviews are intended to generate standardised data using closed questions and are expected to provide little opportunity for reflection or conversation. The process of being ‘interviewed’ by a stranger, and the content of standardised measures generate unusual experiences for people and would not normally be considered a way of investigating the lived experience of someone caring for a relative with dementia. However, in piloting the adapted embedded method, data was collected revealing the experiences of living with dementia. Although structured interviews are not planned to prompt reflection, participants’ responses suggested that the process of answering such questions in conversation often provided opportunities for them to ‘think aloud’ their own perspectives on dementia. At times, carers disagreed with the wording of the standardised measures and challenged some of the underlying assumptions that were portrayed, such as life as ‘burdensome’ or ‘difficult’. The findings relating to conflicting emotions, desire for normality and determination to preserve agency that form the basis of this discussion resonate with the types of evidence emerging elsewhere in this field, suggesting that the views expressed reflect an accurate picture of the lived experience of some carers.

Carers in this study clearly expressed their intentions to maintain agency and enable ‘normality’ within the confines of dementia. They linked this to a desire to arrange care that fulfilled the families’ expectations for compassionate and convenient provision. This suggests that, for some carers, their belief in themselves and their ability to achieve desired ends persists despite the challenges of declining health, indicating how they maintained a sense of self-efficacy [41]. Self-efficacy, the belief in oneself and one’s ability to achieve desired ends, is thought to influence an individual’s outlook and approach to problems, prompting optimistic or pessimistic attitudes, and plays an important part in carers’ responses to the challenges of dementia [42–45].

Analysing the unsolicited comments assisted interpretation of contradictory responses over the course of the interview, capturing the complexity of people’s emotions and experiences. Carers reacted to the interview schedule in places where the emphasis was negative, for example deploying terms such as ‘burden’ with more positive responses. However, we remain uncertain how the wording of formal questions influences people’s response.

Carers often qualified their responses to articulate complex emotions of loyalty and love and to express the more positive qualities stemming from their role as carers, which could include a new or renewed closeness in their relationship with their relative. Carbonneau and colleagues [46] refer to carers generating ‘enrichment events’ prompted by feelings of self-efficacy. This is illustrated in our findings by carers’ comments conveying contentment and explaining how they maximised the enjoyable moments, valuing what remained of their usual lives, rather dwelling on their losses. These findings correspond with the growing awareness in the literature that positive emotions can co-exist with negative ones and that carers can see caring as positive [44]. The focus on negative scenarios, in the structured interviews, using wording that problematised people’s experience, may have prompted carers’ to critically consider their applicability to their own caring situation.

There were many examples where the unsolicited comments revealed a complex set of emotions and experiences. For instance, the GHQ12 [24] includes questions such as: “In the last few weeks have you felt that you couldn’t overcome your difficulties?” and the ZBI [26] asks whether carers feel they have lost control over their lives. These often elicited unsolicited comments about balancing external care with their own needs, referring to awkward and difficult situations in striving to integrate support into their lives. Many carers described coordinating care as a source of tension, recognised in previous research that found effective collaboration between formal and informal carers difficult to achieve [47, 48]. Nevertheless, the evidence also revealed carers’ self-efficacy in organising care, taking control and blending care to suit the particular family situation, based on negotiating provision, positive personal relationships with formal carers and asserting the importance of relational aspects of care.

In attending directly to the voices of carers, as well as through their responses to standardised measures, we see a counterbalancing emphasis on the mixed emotions of carers, where they frequently qualify the answers they provide in response to interview questions. Furthermore, the embedded method enabled carers to express the complexity of practical arrangements: carers responded to questions that aimed to quantify the amount of support they received by expanding on the adequacy of formal support and outlining the dilemmas of maintaining ‘life as usual’ when managing care arrangements. These findings reiterate the importance of wellbeing, prioritised by the Care Act in England [49] which introduced the principle of ‘wellbeing’ incorporating the concept of individuals having control over their day-to-day life, including over care and support. This is now a principal duty of local government when designing services.

### Evaluating the method

Trialling an adapted embedded qualitative method demonstrated the potential for enriching data generated from a quantitative study. It revealed the complexity of the lived experiences that are difficult to capture using standardised measures but which participants are keen to articulate themselves. The evidence suggests that the data was detailed enough to be analysed using qualitative methods, to reveal the ‘lived experience’ of participants. Moreover, it fulfilled the intention to minimise the participant burden, by harvesting data that occurs naturally, but is not part of the quantitative analysis. The importance of minimising harm and including the voice of those experiencing dementia has been reiterated by researchers [50, 51] but, to our knowledge, there has been little discussion about how to conduct research that will keep the burden on families to a minimum.

As anticipated, the method gave rise to new challenges that require further consideration. Issues about the nature of the relationship between the qualitative findings and the structured measures will be considered in more depth in future evaluations of the adapted embedded method. Areas for further investigation include how standardised measures influence unsolicited comments and how qualitative findings can be integrated with quantitative results. The following observations arising from the findings are intended to promote debate, discussion and further research.

First, the interactions involved in using standardised processes and measures are likely to vary in the real world research encounter. Whilst interviewers may aim to adopt a standard process of questioning that is consistent between interviews and does not include prompting or general conversation, participants themselves are likely to vary in how readily or comfortably they adhere to this process or feel free to qualify or add comments. There are also likely to be differences in interviewer technique which may influence the extent to which participants expand their answers.

Second, the standardised tools employed in quantitative studies intended to measure wellbeing and health are necessarily focused on difficulties and challenges for people, using words that do not necessarily align with people’s outlook on life. Questions such as ‘how burdened’ or ‘how worried’ often prompted participants to qualify their answers or to reword the question. The wording of the questions is potentially loaded and may have influenced the carer, either to concur with the loading or refute it more strenuously. There were also occasions where the question options may have constrained the range of possible responses from carers, when they looked to the interviewer to “help them out” and where subsequent questioning by the interviewer, may have resulted in them giving an answer that did not closely reflect their experience.

Third, in analysing the data it was shown that, although the interview structure sought to compartmentalise experiences and feelings, for example into ‘service provision’ or ‘carer burden’, comments relating to these and other themes nonetheless continued to emerge throughout the interviews, revealing relatively little containment. This confirms the assumption that participants would contribute conversationally throughout the interview yielding results that went beyond the scope of the formally structured parts.

Fourthly, given that research in the field of dementia is complex, a mixed-methods approach which can recognise and synthesise both quantitative and qualitative data offers great potential for improving our understanding of what issues of living with dementia are especially relevant and why [52–55]. Adapting the embedded qualitative method to enable data collection as part of the structured interview within a larger quantitative study could prove an efficient means to explore the lived experience. The current study also demonstrated the bonus of exploring how people respond interactively during structured interviews.

The findings do not allow us to draw firm conclusions about the influence of structured questions or to define the nature of a formal interview but prompt us to consider how we could re-examine the complex social context of research encounters more carefully, in effect considering the ‘lived experience’ of completing standardised measures of health and wellbeing. Recognising the benefits of analysing unsolicited comments generated during a structured interview highlights the importance of maximising opportunities to explore individual experiences, endorsing the inclusion of adapted embedded qualitative methods.

## Conclusions

Qualitative methods are increasingly included in studies to provide a richer understanding of people’s experience. This paper has examined the potential of an adapted embedded qualitative method to enrich a dataset generated by using standardised research measures without increasing the research burden on participants or interviewers and has foregrounded the importance of evidence made available in this way about carers’ experience of living with dementia and participating in the research process. The substantive findings contain valuable information for planning and delivery of services for people in later stage dementia and their carers in the future. The methodological findings hold some promise for the positive capacity of the method. Further testing in other studies will be required to ascertain more about the nature of its efficacy in other contexts as well as how the specific findings can be used to enrich the understanding of the larger study aims.

## Additional file

Additional file 1: Carer Questionnaire. (DOC 367 kb)

## Data Availability

Supporting data can be obtained on request from the corresponding author.

## Abbreviations

GHQ12: General Health Questionnaire
PANT: The Practitioner Assessment of Network Type
PPCI: Public, Patient, and Carer Involvement
TIDE: Together in Dementia Everyday
ZBI: Zarit Burden Interview
