# Supplementary material for: Applying a new concept of embedding qualitative research: an example from a quantitative study of carers of people in later stage dementia
Source: BMC Geriatr. 2019 Aug 22;19:227. doi: 10.1186/s12877-019-1240-x (PMC6704652; doi:10.1186/s12877-019-1240-x)
Supplement: Supplementary file 1 — Carer Questionnaire. (DOC 367 kb) [file 12877_2019_1240_MOESM1_ESM.doc]

**CARER QUESTIONNAIRE**

**(Edited to comply with copyright)**

**Background information**

1. **Gender of carer**

☐ Male ☐ Female

1. **How old were you at your last birthday?** ……….years
2. **What is your ethnic group? Choose one option that best describes your ethnic group or background.**

| **White:** |  | |
| --- | --- | --- |
| English/Welsh/Scottish/Northern Irish/British | **☐** | |
| Irish | **☐** |  |
| Gypsy or Irish Traveller | **☐** |  |
| Other White background, please describe_______________________ | **☐** |  |
| **Mixed/Multiple ethnic groups:** |  |  |
| White and Black Caribbean | **☐** |  |
| White and Black African | **☐** |  |
| White and Asian | **☐** |  |
| Other Mixed/Multiple ethnic background, please describe___________ | **☐** |  |
| **Asian/Asian British:** |  |  |
| Indian | **☐** |  |
| Pakistani | **☐** |  |
| Bangladeshi | **☐** |  |
| Chinese | **☐** |  |
| Other Asian background, please describe______________________ | **☐** |  |
| **Black/African/Caribbean/Black British:** |  |  |
| African | **☐** |  |
| Caribbean | **☐** |  |
| Other Black/African/Caribbean background, please describe________ | **☐** |  |
| **Other ethnic group:** |  |  |
| Arab | **☐** |  |
| Any other ethnic group, please describe________________________ | **☐** |  |

1. **What is your marital status?**

| Single | **☐** |
| --- | --- |
| Married or cohabiting | **☐** |
| Separated | **☐** |
| Divorced | **☐** |
| Widowed | **☐** |

1. **What is your relationship to the person you care for …I am their:**

**…………………………………………………**

| Spouse/partner | **☐** |
| --- | --- |
| Parent | **☐** |
| Grandchild | **☐** |
| Son/daughter | **☐** |
| Brother/sister | **☐** |
| Other relative | **☐** |
| Friend | **☐** |
| Paid carer | **☐** |
| Other (*please specify*) | **☐** |

**6. Where does your relative/friend usually live?**

…………………………………………………………

| With you | **☐** |
| --- | --- |
| Living in own home – with relative | **☐** |
| Living in own home – alone | **☐** |
| Supported accommodation, e.g. sheltered | **☐** |
| Residential care *(not permitted at interview 1)* | **☐** |
| Nursing home care *(not permitted at interview 1)* | **☐** |
| Other (e.g. 24 hour live in carers) (*please specify*) ……………………… | **☐** |

**7. Does your relative/friend live in…?**

| Owner occupied flat or house | **☐** |
| --- | --- |
| Privately rented flat or house | **☐** |
| Rented from local authority or housing association/cooperative | **☐** |
| Other (*please specify*) ……………………… | **☐** |

**8. Has s/he lived anywhere else in the *last six months*?** ☐ Yes ☐ No

**If yes, please indicate number of days spent living there (exclude hospital stays).**

|  | Please tick all that apply | Number of days in last six months |
| --- | --- | --- |
| Residential care |  |  |
| Nursing home care |  |  |
| Supported accommodation, e.g. sheltered |  |  |
| Other (*please specify*) |  |  |
| Other (*please specify*) |  |  |

1. **I would like to ask you some questions about your relative’s/friend’s contact with family/friends**

**Example question:**

| **Question** | **Response categories** | **Tick** | *Office use only** |
| --- | --- | --- | --- |
| **How often does he/she see any of his/her children or other relatives to speak to?** | Never/no relative | ☐ | *A* |
|  | Daily | ☐ | *B* |
|  | 2-3 times a week | ☐ | *C* |
|  | At least weekly | ☐ | *D* |
|  | At least monthly | ☐ | *E* |
|  | Less often | ☐ | *F* |
|  | Yes, occasionally | ☐ | *B* |
|  | No | ☐ | *C* |

From: PANT Instrument Wenger © 2002 (Omitted for copyright reasons)

1. **On a typical day during the *last six months*, how much time did you spend assisting your relative/friend with the following tasks? Record 0 if no time spent.**

|  | **Number of…** | |
| --- | --- | --- |
| **Type of support** | **Hours per day** | **Days per week** |
| Toilet visits, eating, dressing, grooming, walking and bathing |  |  |
| Shopping, food preparation, housekeeping, laundry, transportation, taking medication, and managing financial matters |  |  |
| Supervising your relative/friend (that is, preventing dangerous events) |  |  |

**11a. In addition to your caring role, please tell us which of the following also applies to you?**

| Retired | **☐** |
| --- | --- |
| Employed full-time | **☐** |
| Employed part-time (working 30 hours or less) | **☐** |
| Self-employed full-time | **☐** |
| Self-employed part-time | **☐** |
| Not in paid work | **☐** |
| Doing voluntary work | **☐** |
| Other (*please specify*) ……………………… | **☐** |

**11b. How many hours, on average, do you work for pay per week? (record 0 if none)**

…………hours per week ***If ‘0’ at Q11b skip to Q13***

**11c. Of these hours are you for some part paid to care for your relative/friend?**

Yes ☐

No ☐

**11d. If yes, how many hours per week? (record 0 if none)**

…………hours per week

**12a. During the *last six months*, have you needed to cut down the number of hours that you usually work because of your caring responsibilities?**

Yes ☐

No ☐

**12b. If yes, how many hours per week?**

…………hours per week

**13. During the *last six months*, please specify the number of times that your caring responsibilities affected your work in the following ways:**

Number of times (0 if none)

13a Missed a **whole** day of work .…………

13b Missed a **part** of a day of work ………….

**Carer Health and Well-Being**

**Could I please ask you some questions about your health?**

**14. Please indicate the ONE box that best describes your health TODAY**

**Example question:**

| **Usual activities** *(e.g. work, study, housework, family or leisure activities)* |  |
| --- | --- |
| I have no problems doing my usual activities |  |
| I have slight problems doing my usual activities |  |
| I have moderate problems doing my usual activities |  |
| I have severe problems doing my usual activities |  |
| I am unable to do my usual activities |  |
| I have severe pain or discomfort |  |
| I have extreme pain or discomfort |  |

From: UK (English) © 2009 EuroQol Group EQ-5D™ is a trade mark of the EuroQol

Group (Omitted for copyright reasons)

**15. We should like to know if you have had any medical complaints, and how your health has been in general, over the past few weeks. Please answer ALL the questions simply by selecting the answer which you think most nearly applies to you. Remember that we want to know about present and recent complaints, not those that you had in the past. It is important that you try to answer ALL the questions [these can be self-completed if carer prefers].**

**Have you recently …**

**Example question:**

| Felt capable of making decisions about things? | More so than usual | Same as usual | Less so than usual | Much less capable |
| --- | --- | --- | --- | --- |
|  |  |  |  |

From: GHQ-12 **©** Goldberg (1978). (Omitted for copyright reasons)

**16. The following is a list of statements which reflect how people sometimes feel when taking care of another person. After each statement, please indicate how often you feel that way. There are no right or wrong answers.**

**Example question:**

|  | 0 | 1 | 2 | 3 | 4 |
| --- | --- | --- | --- | --- | --- |
|  | Never | Rarely | Sometimes | Quite frequently | Nearly always |
| Do you feel that your relative currently affects your relationship with other family members or friends in a negative way? |  |  |  |  |  |

Carer burden © Zarit and Zarit (1983, 1990). SSCQ © Vernooij-Dassen et al. (1999). (Omitted for copyright reasons)

**Health and Well-Being of Your Relative/Friend**

**17. I would like to ask you about your relative’s/friend’s life, as you are the person who knows him/her best. There are no right or wrong answers. Just give the answer that best describes how your relative/friend** **has felt in the last week. If possible try and give the answer that you think your relative/friend** **would give. Don’t worry if some questions appear not to apply to your relative/friend***.* **We have to ask the same questions of everybody.**

**For all the questions I’m going to ask you, I want you to think about the last week.**

**First I’m going to ask you about your relative’s/friend’s feelings. In the last week, would you say that your relative/friend has felt…**

Instruction to interviewer: Read each of the following questions verbatim and show the respondent the appropriate response card (SHOW CARD 3A for items 1-31 and SHOWCARD 3B for item 32).

**Example question:**

|  | A lot | Quite a bit | A little | Not at all |
| --- | --- | --- | --- | --- |
| 1. Cheerful? |  |  |  |  |

DEMQOL-PROXY version 4 **©** Institute of Psychiatry, King’s College London (2011). (Omitted for copyright reasons)

**18. I’d like to ask you some questions about the things your relative/friend does in their everyday life. Thinking of the last 2 weeks, tick the box that represents your relative's/friend's ability:**

(If in doubt about which box to tick, choose the level of ability which represents their *average* performance over the last two weeks)

|  | (0) | (1) | (2) | (3) |  |
| --- | --- | --- | --- | --- | --- |
| 1. **Food** | Selects and prepares food as required | Able to prepare food if ingredients set out | Can prepare food if prompted step by step | Unable to prepare food even with prompting and supervision | Not applicable |
|  |  |  |  |  |

From: BADLS © Bucks et al. (1996) (Omitted for copyright reasons)

**19a. I’d like to ask you what kinds of help you (the carer) or your relative/friend have received over the *last six months at home*? You may not have received any of the following types of help but just try and answer from your experience** Instruction to interviewer: please read out list of workers/services in column 1 and indicate if they have received the service).

**19b. For each type of care/service received please also specify the number of visits and how long the visit is on average? If both carer and relative/friend receive a service please indicate their respective inputs.**

**19c. For each type of care/service please indicate, by placing the relevant letter(s) in the final column, *what was done* by the worker?**

**Instruction to interviewer: where a component is provided by a worker/service not listed, please add them to ‘other’ and complete the columns**

| **Type of worker/service** | **Please tick if received** | | **Number of visits within last 6 months** | | **Average duration of visit in minutes** | | **What was done [please indicate all that apply – see SHOW CARD 5 for identifier codes]** |
| --- | --- | --- | --- | --- | --- | --- | --- |
|  | **You**  **(carer)** | **Your relative** | **You**  **(carer)** | **Your relative** | **You**  **(carer)** | **Your relative** |  |
| Home care worker |  |  |  |  |  |  |  |
| Support worker |  |  |  |  |  |  |  |
| Care/case manager |  |  |  |  |  |  |  |
| Community mental health nurse |  |  |  |  |  |  |  |
| Occupational therapist |  |  |  |  |  |  |  |
| Admiral nurse |  |  |  |  |  |  |  |
| Dementia advice worker |  |  |  |  |  |  |  |
| Counsellor |  |  |  |  |  |  |  |
| Community/district nurse |  |  |  |  |  |  |  |
| Social worker |  |  |  |  |  |  |  |
| Voluntary organisation worker (e.g. Age UK, Alzheimer’s Society) |  |  |  |  |  |  |  |
| Other ***(****please specify)* |  |  |  |  |  |  |  |
| Other ***(****please specify)* |  |  |  |  |  |  |  |

**20a. What other services have you or your relative/friend received over the *last six months* including visits to surgeries, clinics and centres? Please do not include anything already recorded above in question 19.** Instruction to interviewer: please read out list of workers/services in column 1 and indicate if they have received the service.

**20b. For each type of care/service received please also specify the number of visits and how long the visit is on average? If both carer and relative/friend receive a service please indicate their respective inputs.**

| **Type of worker/service** | **Please tick if received** | | **Number of visits within last 6 months** | | **Average duration of visit in minutes** | |
| --- | --- | --- | --- | --- | --- | --- |
|  | **You**  **(carer)** | **Your relative** | **You**  **(carer)** | **Your**  **relative** | **You**  **(carer)** | **Your**  **relative** |
| General practitioner (inc. telephone consultations) |  |  |  |  |  |  |
| GP practice nurse |  |  |  |  |  |  |
| Community pharmacist |  |  |  |  |  |  |
| Geriatrician |  |  |  |  |  |  |
| Neurologist |  |  |  |  |  |  |
| Psychiatrist |  |  |  |  |  |  |
| Psychologist |  |  |  |  |  |  |
| Physiotherapist |  |  |  |  |  |  |
| Dietician |  |  |  |  |  |  |
| Health visitor |  |  |  |  |  |  |
| Chiropodist/podiatrist |  |  |  |  |  |  |
| Benefits advisor (e.g. Citizens Advice Bureau) |  |  |  |  |  |  |
| Transportation (e.g. door-to-door accessible minibus service such as Ring and Ride) |  |  |  |  |  |  |
| Drop in centre |  |  |  |  |  |  |
| Home delivered meals |  |  |  |  |  |  |
| **Type of worker/service** | **Please tick if received** | | **Number of visits within last 6 months** | | **Average duration of visit in hours** | |
|  | **You**  **(carer)** | **Your relative** | **You**  **(carer)** | **Your**  **relative** | **You**  **(carer)** | **Your**  **relative** |
| Day care centre (including respite day care) |  |  |  |  |  |  |
| Short-term respite care in residential/nursing home |  |  |  |  |  |  |
| Other *(please specify)* |  |  |  |  |  |  |
| Other *(please specify)* |  |  |  |  |  |  |

**21a. What hospital (inpatient and outpatient) services have you or your relative/friend received over the *last six months*?**

**21b. For each service, please provide the total number of admissions/visits and total length of stay**

| **Service** | **Number of admissions/visits in total** | | **How long in total (total number of days)** | |
| --- | --- | --- | --- | --- |
| **Inpatient (24 hours or more)** | **You**  **(carer)** | **Your relative** | **You**  **(carer)** | **Your relative** |
| Accident and emergency (ward admission) |  |  |  |  |
| Geriatric ward |  |  |  |  |
| Acute psychiatric ward |  |  |  |  |
| General medical ward |  |  |  |  |
| Surgical ward |  |  |  |  |
| Rehabilitation ward/facility |  |  |  |  |
| Long-stay ward |  |  |  |  |
| Other inpatient visit (*please specify*) |  |  |  |  |
| Other inpatient visit (*please specify*) |  |  |  |  |
| Other inpatient visit (*please specify*) |  |  |  |  |
| **Outpatient (less than 24 hours)** | **Number of visits in total** | | **How long in total (total number of hours)** | |
|  | **You**  **(carer)** | **Your relative** | **You**  **(carer)** | **Your relative** |
| Accident and emergency visit |  |  |  |  |
| Psychiatric outpatient visit |  |  |  |  |
| Day hospital (excl. regular day activities) |  |  |  |  |
| Other outpatient visit (*please specify*) |  |  |  |  |
| Other outpatient visit (*please specify*) |  |  |  |  |
| Other outpatient visit (*please specify*) |  |  |  |  |

**22. Have you or your relative/friend received any adaptations or equipment to help you/them to live independently?** Instruction to interviewer: read list (record all that apply; if none state ‘none’).

| **Adaptations,**  **Equipment and products** | **Type of adaptation or equipment *(list all)*** | **Who / what organisation paid for this?** |
| --- | --- | --- |
| Alterations to your/their home  *e.g. putting in shower cubicle, downstairs toilet, wash room, stair lift, outside key box* | (1)........................................................................  (2)........................................................................  (3)........................................................................  (4)........................................................................  (5)........................................................................ | (1)...................................  (2)...................................  (3)....................................  (4)..................................  (5).................................. |
| Equipment  *(e.g. grab rails, raised toilet, bath seat)* | (1)........................................................................  (2)........................................................................  (3)........................................................................  (4)........................................................................  (5)........................................................................ | (1)...................................  (2)...................................  (3)....................................  (4)..................................  (5).................................. |
| Assistive technology  *(e.g. sensors, gas alarms, automatic lighting)* | (1)........................................................................  (2)........................................................................  (3)........................................................................  (4)........................................................................  (5)........................................................................ | (1)...................................  (2)...................................  (3)....................................  (4)..................................  (5).................................. |

**23. Have you or your relative/friend used an ambulance in the last six months?** Instruction to interviewer: count outward and return journey as separate trips. ☐ Yes ☐ No

|  | **You**  **(carer)** | **Number of times** | **Your relative** | **Number of times** |
| --- | --- | --- | --- | --- |
| Emergency |  |  |  |  |
| Routine transport |  |  |  |  |

**24. Are the services that you or your friend/relative has received** funded by (tick all that apply):

|  | **You**  **Carer** | **Your relative** | **Don’t know** |
| --- | --- | --- | --- |
| Your friend/relative/you |  |  |  |
| The local authority |  |  |  |
| The NHS |  |  |  |
| A personal budget received as a direct payment |  |  |  |
| Continuing healthcare |  |  |  |
| Attendance allowance |  |  |  |
| Other *(please specify)* |  |  |  |

**Help from Health and Social Care Services**

**25. The following questions relate to any help with daily tasks your relative/friend may have received over the last six months.**

**25.1** Rising and retiring (e.g. getting into or out of bed, washing self (not bathing) and dressing)

| **1.1 Can your relative/friend RELY on someone being able to help him/her? Is the care….** |  |
| --- | --- |
| Reliable always |  |
| Usually reliable, but sometimes lapses |  |
| Reliable only at certain times e.g. not at weekends/nights |  |
| Doubtful/ mixed |  |
| Unreliable/unpredictable |  |
| Not reliable, but person not reliant upon help |  |
| No answer/not applicable |  |
| **1.2 Is the help really EFFECTIVE? Is it done/given in the way in which he/she would like? Probe**. |  |
| Yes, definitely |  |
| Usually, but sometimes lapses |  |
| Ineffective at specific times e.g. weekends/nights |  |
| Doubtful/ mixed |  |
| Ineffective |  |
| No answer/not applicable |  |
| **1.3 Is he/she getting as much help as he/she needs? Is it SUFFICIENT? Probe**. |  |
| Yes, definitely |  |
| Usually, but sometimes varies |  |
| Insufficient at specific times e.g. weekends/nights |  |
| Doubtful/ mixed |  |
| Extra essential |  |
| No answer/not applicable |  |

**25.2 Personal care** (e.g. feeding, rising from chair, toileting, grooming, bathing, medication management, self-monitoring (avoid risk of self-neglect))

| **2.1 Can your relative/friend RELY on someone being able to help him/her? Is the care….** |  |
| --- | --- |
| Reliable always |  |
| Usually reliable, but sometimes lapses |  |
| Reliable only at certain times e.g. not at weekends/nights |  |
| Doubtful/ mixed |  |
| Unreliable/unpredictable |  |
| Not reliable, but person not reliant upon help |  |
| No answer/not applicable |  |
| **2.2 Is the help really EFFECTIVE? Is it done/given in the way in which he/she would like? Probe**. |  |
| Yes, definitely |  |
| Usually, but sometimes lapses |  |
| Ineffective at specific times e.g. weekends/nights |  |
| Doubtful/ mixed |  |
| Ineffective |  |
| No answer/not applicable |  |
| **2.3 Is he/she getting as much help as he/she needs? Is it SUFFICIENT? Probe**. |  |
| Yes, definitely |  |
| Usually, but sometimes varies |  |
| Insufficient at specific times e.g. weekends/nights |  |
| Doubtful/ mixed |  |
| Extra essential |  |
| No answer/not applicable |  |

**25.3 Daily Domestic Care (e.g. light housework, preparation of meals, drinks or snacks)**

| **3.1 Can your relative/friend RELY on someone being able to help him/her? Is the care….** |  |
| --- | --- |
| Reliable always |  |
| Usually reliable, but sometimes lapses |  |
| Reliable only at certain times e.g. not at weekends/nights |  |
| Doubtful/ mixed |  |
| Unreliable/unpredictable |  |
| Not reliable, but person not reliant upon help |  |
| No answer/not applicable |  |
| **3.2 Is the help really EFFECTIVE? Is it done/given in the way in which he/she would like? Probe**. |  |
| Yes, definitely |  |
| Usually, but sometimes lapses |  |
| Ineffective at specific times e.g. weekends/nights |  |
| Doubtful/ mixed |  |
| Ineffective |  |
| No answer/not applicable |  |
| **3.3 Is he/she getting as much help as he/she needs? Is it SUFFICIENT? Probe**. |  |
| Yes, definitely |  |
| Usually, but sometimes varies |  |
| Insufficient at specific times e.g. weekends/nights |  |
| Doubtful/ mixed |  |
| Extra essential |  |
| No answer/not applicable |  |

**25.4** Weekly Domestic Care (e.g. heavy housework (cleaning), shopping, laundry, soiled linen)

| **4.1 Can your relative/friend RELY on someone being able to help him/her? Is the care….** |  |
| --- | --- |
| Reliable always |  |
| Usually reliable, but sometimes lapses |  |
| Reliable only at certain times e.g. not at weekends/nights |  |
| Doubtful/ mixed |  |
| Unreliable/unpredictable |  |
| Not reliable, but person not reliant upon help |  |
| No answer/not applicable |  |
| **4.2 Is the help really EFFECTIVE? Is it done/given in the way in which he/she would like? Probe**. |  |
| Yes, definitely |  |
| Usually, but sometimes lapses |  |
| Ineffective at specific times e.g. weekends/nights |  |
| Doubtful/ mixed |  |
| Ineffective |  |
| No answer/not applicable |  |
| **4.3 Is he/she getting as much help as he/she needs? Is it SUFFICIENT? Probe**. |  |
| Yes, definitely |  |
| Usually, but sometimes varies |  |
| Insufficient at specific times e.g. weekends/nights |  |
| Doubtful/ mixed |  |
| Extra essential |  |
| No answer/not applicable |  |

Help with activities of daily living © Challis and Davies (1986).
